# Impact of a goal-directed factor-based coagulation management on thromboembolic events following major trauma

**DOI:** 10.1186/s13049-019-0697-0

**Published:** 2019-12-30

**Authors:** Anais L. Stein, Julian Rössler, Julia Braun, Kai Sprengel, Patrick E. Beeler, Donat R. Spahn, Alexander Kaserer, Philipp Stein

**Affiliations:** 1 Department of Forensic Medicine and Imaging, Institute of Forensic Medicine, University of Zurich, Winterthurerstrasse 190/52, 8057 Zurich, Switzerland; 2 Institute of Anaesthesiology, University and University Hospital Zurich, Raemistrasse 100, 8091 Zurich, Switzerland; 3 Departments of Biostatistics and Epidemiology, Epidemiology, Biostatistics and Prevention Institute, University of Zurich, Hirschengraben 84, 8001 Zurich, Switzerland; 4 Department of Trauma, University and University Hospital Zurich, Raemistrasse 100, 8091 Zurich, Switzerland; 5 Department of Internal Medicine, University Hospital Zurich, Raemistrasse 100, 8091 Zurich, Switzerland; 6 Institute of Anaesthesiology, Cantonal Hospital Winterthur, Brauerstrasse 15, 8400 Winterthur, Switzerland

**Keywords:** Coagulation management, Coagulation factors, Thromboembolic events, Transfusion, Trauma

## Abstract

**Background:**

A factor-based coagulation management following major trauma is recommended as standard of care by the European Trauma Treatment Guidelines. However, concerns about the thromboembolic risk of this approach are still prevalent. Our study therefore aims to assess if such a haemostatic management is associated with an increased risk for thromboembolic events.

**Methods:**

In this retrospective observational study carried out at the University Hospital Zurich we compared two three-year periods before (period 1: 2005–2007) and after (period 2: 2012–2014) implementation of a factor-based coagulation algorithm. We included all adult patients following major trauma primarily admitted to the University Hospital Zurich. Thromboembolic events were defined as a new in-hospital appearance of any peripheral thrombosis, arterial embolism, pulmonary embolism, stroke or myocardial infarction. A logistic regression was performed to investigate the association of thromboembolic events with possible confounders such as age, sex, specific Abbreviated Injury Scale (AIS) subgroups, allogeneic blood products, and the coagulation management.

**Results:**

Out of 1138 patients, 772 met the inclusion criteria: 344 patients in period 1 and 428 patients in period 2. Thromboembolic events were present in 25 patients (7.3%) of period 1 and in 42 patients (9.8%) of period 2 (raw OR 1.39, 95% CI 0.83 to 2.33, *p* = 0.21). Only AIS extremities (adjusted OR 1.26, 95% CI 1.05 to 1.52, *p* = 0.015) and exposure to allogeneic blood products (adjusted OR 2.39, 95% CI 1.33 to 4.30, *p* = 0.004) were independently associated with thromboembolic events in the logistic regression, but the factor-based coagulation management was not (adjusted OR 1.60, 95% CI 0.90–2.86, *p* = 0.11).

**Conclusion:**

There is no evidence that a goal-directed, factor-based coagulation management is associated with an increased risk for thromboembolic events following major trauma.

## Background

A goal-directed, factor-based coagulation management following major trauma is recommended as standard of care by the European Trauma Treatment Guidelines to treat and prevent trauma induced coagulopathy [1]. Such individualized coagulation management needs to be guided by viscoelastic testing and laboratory values [2, 3] to meet patients’ demands and is therefore more complex than traditional transfusion strategies of red blood cells (RBC), fresh frozen plasma (FFP) and platelet concentrates (PC) at fixed ratios [4]. Predefined coagulation algorithms were introduced to tackle this issue and to guide the haemostatic management of clinicians at the emergency department [5, 6]. Such coagulation algorithms were proven to reduce the incidence of massive transfusion [7], the transfusion of allogeneic blood products [3, 5, 7] and to improve survival rate [6] of patients following major trauma. Beneficial effects of a factor-based coagulation management have been reported for patients following trauma, as well as in the early identification and individualized treatment of coagulopathy in major obstetric haemorrhage [8]. Moreover, in patients undergoing cardiac surgery a point of care coagulation management reduced the exposure to allogeneic blood products, lowered the re-exploration rate and decreased the incidence of postoperative acute kidney injury as well as thromboembolic events [9]. It was shown that a goal-directed factor concentrate based coagulation and transfusion management compared to a fixed ratio transfusion approach reduced the incidence of massive transfusion and the exposure of patients to allogeneic blood products [3, 7]. Moreover, 24 h and in-hospital mortality were significantly reduced [7]. However, concerns do remain about the thromboembolic risk of the factor-based resuscitation approach in trauma patients.

Our study therefore aims to assess if such a haemostatic management is associated with an increased risk for thromboembolic events following major trauma.

## Methods

### Study design & participants

We conducted a retrospective cohort study comparing two time periods with different transfusion and coagulation management strategies of trauma patients in a single, tertiary care hospital with a level-1 trauma centre. As changes to the transfusion and coagulation management protocol were gradually implemented from 2008 until 2012, we investigated two three-year periods: The first from 2005 until 2007 before and the second one after the implementation from 2012 until 2014. In these two periods we included all severely injured trauma patients aged ≥16 with an injury severity score (ISS) ≥ 16, who were primarily admitted to the University Hospital of Zurich in Switzerland. We excluded patients with missing or incomplete records as well as patients referred from another hospital.

This study was approved by the local ethics committee (KEK-ZH 2015–0309) and follows the Strengthening the Reporting of Observational Studies in Epidemiology (STROBE) recommendations for cohort studies.

### Setting

As one of the 12 level-1 trauma centres in Switzerland, the University Hospital Zurich treats trauma patients in a highly standardized approach. Specific measures were introduced in the time between the two analysed cohorts. The goal-directed and factor concentrate based coagulation algorithm was used for transfusion and coagulation management in the latter period while in the first period, RBC, FFP and PC were transfused without a goal-directed management. The transfusion and coagulation algorithm is a stepwise guidance for the treatment of all bleeding patients in the University Hospital Zurich and has been described previously in detail by Stein et al. [7]. In period 2, tranexamic acid was applied empirically to patients at risk of significant bleeding analogue to the CRASH-2 trial. One gram of tranexamic acid was given already at the scene of injury or on admission to emergency department. Additional doses of tranexamic acid were evaluated only after viscoelastic proof of hyperfibrinolysis. In addition to transfusion and coagulation management, further measures like primary whole-body CT scan upon admission, damage-control surgery, restrictive fluid resuscitation with crystalloids and concepts of permissive hypotension were also introduced between the two periods. Guidelines on thrombosis prophylaxis were equivalent between the two periods. Standard thrombosis prophylaxis at the University Hospital Zurich includes the application of low-molecular-weight or unfractionated heparin as soon as the bleeding is controlled. Intermittent pneumatic compression devices were applied in case of contraindication to anticoagulant medication.

An internal trauma database and the anaesthesia protocols (from hospital admission to the intensive care unit) provided information about patient characteristics, injury patterns, applied allogeneic blood products (RBC, FFP and PC), coagulation management (fibrinogen, four-factor prothrombin complex concentrate (PCC), coagulation factor XIII, in-hospital tranexamic acid use) and laboratory values. In addition, all radiology reports and all discharge summaries were screened for the diagnosis of any thromboembolic event (peripheral thrombosis, arterial embolism, pulmonary embolism, stroke, or myocardial infarction) during hospitalization.

### Outcomes

Thromboembolic events were defined as a new in-hospital appearance of any peripheral thrombosis, arterial embolism, pulmonary embolism, stroke or myocardial infarction. The primary outcome was the incidence of thromboembolic events in both observation periods and the identification of potential confounders.

### Statistics

Demographics were displayed as means and standard deviations (SD) or counts (n) and proportions (%). Univariable binomial logistic regressions were calculated for raw odds ratios (OR). A multivariable binomial logistic regression model was fitted to ascertain the effects of age, sex, specific Abbreviated Injury Scale (AIS) subgroups (head, thorax, abdomen and extremities), allogeneic blood products, and the coagulation algorithm on the likelihood that patients suffer from any thromboembolic event. The model fit was assessed using the Hosmer-Lemeshow test. A *p*-value of ≤0.05 was used to define statistical significance. All statistical analyses were performed using SPSS version 25 (IBM Corp., Armonk, NY, USA).

## Results

**Fig. 1:**
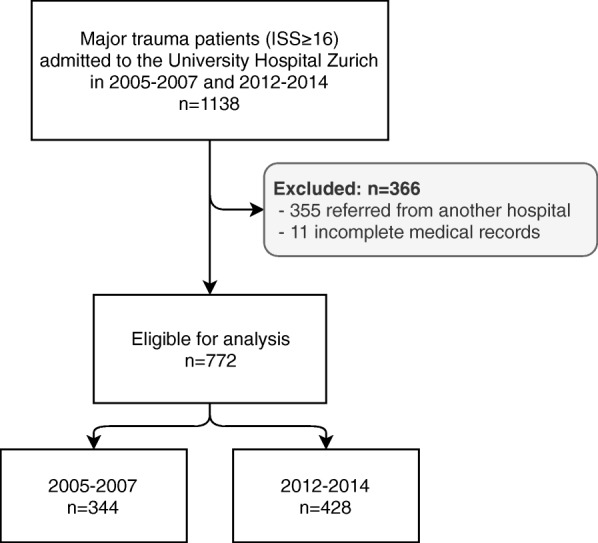
Flowchart of patient selection during the observation period. ISS = Injury Severity Score

We screened 1138 eligible patients (age ≥ 16 years) in period 1 (2005–2007) and period 2 (2012–2014) of which 355 patients were excluded because they were referred from another hospital and 11 patients because of missing emergency department records. The remaining 772 patients were analysed: 344 patients in period 1 and 428 patients in period 2 (Fig. 1). Epidemiologic, demographic and treatment data are presented in Table 1.

**Table 1:** Patients’ characteristics for the period before (Period 1, 2005–2007) and after (Period 2, 2012–2014) the implementation of a goal-directed factor-based coagulation algorithm. Values are means with standard deviations or counts and proportions

	Period 1 (2005–2007) *n* = 344	Period 2 (2012–2014) *n* = 428
Age - years	42.0 (19.0)	51.5 (21.8)
Sex - male	268 (78%)	315 (74%)
Penetrating trauma	29 (8.4%)	16 (3.7%)
Injury severity score	33 (13)	34 (19)
AIS head	3 (2)	3 (2)
AIS thorax	2 (2)	2 (2)
AIS abdomen	1 (2)	1 (1)
AIS extremities	1 (1)	1 (2)
Laboratory values		
Haemoglobin - g/L	111 (29)	107 (53)
Platelet count on entry - 10^3^/mcL	211 (68)	198 (81)
Base Excess on entry - mmol/L	−4.7 (4.6)	−4.4 (5.0)
Lactate on entry - mmol/L	3.2 (2.4)	2.6 (2.4)
Fibrinogen on entry - g/L	1.9 (0.8)	2.2 (0.9)
INR on hospital admission	1.26 (0.5)	1.34 (0.5)
Coagulation factors and allogeneic blood products		
In-hospital TXA	3 (0.9%)	209 (50%)
Fibrinogen	130 (38%)	138 (32%)
PCC	15 (4.4%)	39 (9.1%)
Factor XIII	0	53 (12%)
Transfusion of any allogeneic blood product	181 (53%)	140 (33%)
RBC	175 (51%)	113 (26%)
PC	56 (16%)	66 (15%)
FFP	122 (36%)	67 (16%)

*AIS* Abbreviated Injury Scale, *RBC* Red Blood Cell, *PC* Platelet Concentrate, *FFP* Fresh Frozen Plasma, *PCC* Prothrombin Complex Concentrate, *TXA* Tranexamic Acid

With the implementation of the coagulation algorithm, allogeneic blood transfusions were reduced and the use of factor concentrates increased: 181 (53%) of the patients in period 1 were exposed to any allogeneic blood product transfusion, while only 140 (33%) of the patients were transfused in period 2. The administration of tranexamic acid, four-factor PCC, and coagulation factor XIII increased from 0.9 to 50%, from 4.4 to 9.1% and from 0 to 12% of patients, respectively. Details on the percentage of coagulation factor and blood product use are presented in Table 1.

During period 1, 25 (7.3%) of the patients had a thromboembolic event compared to 42 (9.8%) of the patients in period 2 (raw OR 1.39, 95% CI 0.83–2.33, *p* = 0.21). The detailed comparison of thromboembolic events making up the primary composite outcome are summarized in Table 2 for both periods.

**Table 2:** Incidence of different thromboembolic events and their primary composite endpoint for the period before (Period 1, 2005–2007) and after (Period 2, 2012–2014) implementation of a goal-directed factor-based coagulation algorithm

	Period 1 (2005–2007) *n* = 344	Period 2 (2012–2014) *n* = 428
Peripheral thrombosis	16 (4.7%)	30 (7.0%)
Arterial embolism	3 (0.9%)	1 (0.2%)
Pulmonary embolism	6 (1.7%)	11 (2.6%)
Stroke	1 (0.3%)	2 (0.5%)
Myocardial infarction	0	5 (1.2%)
Thromboembolic events	25 (7.3%)	42 (9.8%)

Values are counts and proportions

The logistic regression model explained 9.0% (Nagelkerke R^2^) of the variance in thromboembolic events. Of the nine predictor variables only two were statistically significant: Injury to the extremities (adj. OR 1.26, 95% CI 1.05 to 1.52, *p* = 0.015) and transfusion of any allogeneic blood product (adj. OR 2.39, 95% CI 1.33 to 4.30, *p* = 0.004, Table 3). The period after the implementation of the goal-directed factor-based coagulation algorithm was not associated with the dependent variable (adj. OR 1.60, 95% CI 0.90–2.86, *p* = 0.11).

**Table 3:** Univariable and multivariable binomial logistic regression for the composite primary outcome of any thromboembolic event. AIS = Abbreviated Injury Scale

	raw OR (95% CI)	*p*-value	adj. OR (95% CI)	*p*-value
Age	1.00 (0.99–1.01)	0.67	1.00 (0.99–1.02)	0.64
Sex - male	1.54 (0.80–2.94)	0.19	1.74 (0.89–3.41)	0.11
AIS head	0.85 (0.75–0.96)	0.01	0.93 (0.80–1.08)	0.32
AIS thorax	1.06 (0.92–1.23)	0.44	0.92 (0.79–1.09)	0.34
AIS abdomen	1.16 (1.01–1.34)	0.042	1.06 (0.90–1.26)	0.48
AIS extremities	1.40 (1.18–1.65)	< 0.001	1.26 (1.05–1.52)	0.015
Any allogeneic blood product	2.57 (1.53–4.30)	< 0.001	2.39 (1.33–4.30)	0.004
Period 2 (2012–2014)	1.39 (0.83–2.33)	0.21	1.60 (0.90–2.86)	0.11

## Discussion

Comparing two periods with a different coagulation management, we found no evidence of increased thromboembolic risk due to a goal-directed, factor-based coagulation algorithm in contrast to haemostatic therapy by means of fixed ratio transfusion of allogeneic blood products. The incidence of the composite outcome of peripheral thrombosis, arterial embolism, pulmonary embolism, stroke, or myocardial infarction did not differ significantly between the two periods. Further, in a multivariable logistic regression adjusting for confounders a factor-based coagulation management period was not associated with an increased risk of thromboembolic events. To the best of our knowledge, this is the first study investigating the impact of a goal-directed factor-based coagulation management on thromboembolic events following major trauma.

Haemostatic resuscitation was traditionally performed by transfusion of RBC, FFP and PC at a fixed ratio [4, 10]. Allogeneic blood transfusions are associated with several adverse events (e.g. infections, volume overload, immunosuppression and kidney injury) [11–14] and it has been shown that a reduced transfusion requirement improved clinical outcomes including mortality [15, 16]. Therefore, a factor-based, goal-directed coagulation management guided by viscoelastic point of care tests [2] has been proposed as a new approach of haemostatic resuscitation [1, 7, 17–19]. This coagulation management was proven to decrease transfusion requirement with beneficial outcomes in trauma patients [1, 7, 17, 18]. The key element of the algorithm is the administration of coagulation factors according to an individualized goal-directed approach based on viscoelastic and laboratory assessment. In our study, period 1 represents the traditional haemostatic management by transfusion of blood products at a fixed ratio. As shown in Table 1, fibrinogen and PCC were also administered in this period but not in a goal-directed fashion guided by viscoelastic testing.

In period 2, after the full implementation, the coagulation and transfusion algorithm was used as the new standard to guide haemostatic therapy. This provides early detection of low fibrinogen levels, low platelet count and the detection of hyperfibrinolysis, all of which can be treated immediately in order to prevent or manage trauma induced coagulopathy [20–24]. Tranexamic acid was used empirically in patients at risk of significant bleeding as investigated in the CRASH-2 trial and not only when hyperfibrinolysis was evident in viscoelastic testing. Since 2013 tranexamic acid was applied to patients already at the scene of injury before reaching the hospital, so our reported (in-hospital) incidence may well be underestimated. This haemostatic approach is a key element in the treatment of major trauma patients and recommended by the European Trauma Treatment Guidelines [1].

Frequency of thrombotic complications in trauma patients was reported in 1.1% up to 34.3% [25–27]. Our incidence of thromboembolic events was below 10% in both periods and thereby in the lower range. There are many possible confounders explaining this broad range of reported incidence. In the era of ultrasound, more thromboembolic events are discovered - occasionally even in asymptomatic patients [28]. Therefore, we assume that the incidence of thromboembolism of period 1 in our study might even be underestimated. Another confounder is the trauma mechanism. While in Europe most patients suffer from blunt injuries, penetrating injuries are leading in the United States [29]. Fractures of extremities are a well-known risk factor for venous thromboembolism. Especially patients suffering from pelvic fractures have a very high risk to develop deep venous thrombosis despite mechanical and pharmaceutical thromboprophylaxis [30]. Consequently, the four AIS subgroups (head, extremities, thorax, abdomen) were chosen to select an adequate number of confounding variables with clinical relevance in regard to the risk of thrombosis. In our multivariable analysis, injuries to extremities were independently associated with the primary outcome. In addition, exposure to allogeneic blood products also proved to be an independent risk factor for thromboembolic events in trauma patients. That finding is congruent to recently published data of 750.937 patients undergoing surgery showing an association of perioperative RBC transfusion with venous thromboembolism [31]. Major trauma patients suffer from extended soft tissue injury and subsequent inflammatory response leading to a diffuse activation of coagulation factors, which culminates in a hypercoagulable state in the post-aggression phase [32]. While this alone increases the risk of developing venous thromboembolism during hospitalization, trauma patients are further exposed to numerous additional risk factors, like prolonged immobilization and a restrictive antithrombotic prophylaxis in case of a traumatic brain injury.

Several limitations regarding our study should be considered in interpreting our findings. Foremost, this was a retrospective observational study and is bound by the inherent limitations of its design. In this sense, we can only deduct association and not causation. Further, there may be some confounders which we cannot detect and correct retrospectively. To limit this, we calculated multivariable models adjusting for possible confounders. Variables of our multivariable regression model were chosen for clinical reasons in order to represent known confounders influencing the incidence of thromboembolic events. Confounders were not selected according to a stepwise variable selection. Additionally, retrospective studies are confined by the amount of available data, impeding sampling for adequate power. Especially in the current study, as we do not have a prespecified equivalence margin, we can only state that we found no evidence for an increased thromboembolic risk, but we cannot definitively exclude a possible effect. Due to the low count of thromboembolic events, we were limited in the analysis to identify confounders in more detail. Future studies are encouraged to build on our work to investigate single factors in more detail.

## Conclusion

There is no evidence that a goal-directed, factor-based coagulation management is associated with an increased risk for thromboembolic events following major trauma.

## Data Availability

All data generated or analysed during this study are included in this published article.

## Abbreviations

FFP: Fresh frozen plasma
GCS: Glasgow coma scale
PC: Platelet concentrate
PCC: Prothrombin complex concentrate
RBC: Red blood cell
TXA: Tranexamic acid
